# RNA‐Seq detects a *SAMD12‐EXT1* fusion transcript and leads to the discovery of an *EXT1* deletion in a child with multiple osteochondromas

**DOI:** 10.1002/mgg3.560

**Published:** 2019-01-10

**Authors:** Gavin R. Oliver, Patrick R. Blackburn, Marissa S. Ellingson, Erin Conboy, Filippo Pinto e Vairo, Matthew Webley, Erik Thorland, Matthew Ferber, Els Van Hul, Ilse M. van der Werf, Wim Wuyts, Dusica Babovic‐Vuksanovic, Eric W. Klee

**Affiliations:** ^1^ Department of Health Sciences Research Mayo Clinic Rochester Minnesota; ^2^ Center for Individualized Medicine Mayo Clinic Rochester Minnesota; ^3^ Department of Laboratory Medicine and Pathology Mayo Clinic Rochester Minnesota; ^4^ Department of Clinical Genomics Mayo Clinic Rochester Minnesota; ^5^ Center of Medical Genetics University of Antwerp and Antwerp University Hospital Antwerp Belgium

**Keywords:** exostoses, gene fusion, multiple hereditary, osteochondroma

## Abstract

**Background:**

We describe a patient presenting with pachygyria, epilepsy, developmental delay, short stature, failure to thrive, facial dysmorphisms, and multiple osteochondromas.

**Methods:**

The patient underwent extensive genetic testing and analysis in an attempt to diagnose the cause of his condition. Clinical testing included metaphase karyotyping, array comparative genomic hybridization, direct sequencing and multiplex ligation‐dependent probe amplification and trio‐based exome sequencing. Subsequently, research‐based whole transcriptome sequencing was conducted to determine whether it might shed light on the undiagnosed phenotype.

**Results:**

Clinical exome sequencing of patient and parent samples revealed a maternally inherited splice‐site variant in the doublecortin (*DCX*) gene that was classified as likely pathogenic and diagnostic of the patient's neurological phenotype. Clinical array comparative genome hybridization analysis revealed a 16p13.3 deletion that could not be linked to the patient phenotype based on affected genes. Further clinical testing to determine the cause of the patient's multiple osteochondromas was unrevealing despite extensive profiling of the most likely causative genes, *EXT1* and *EXT2*, including mutation screening by direct sequence analysis and multiplex ligation‐dependent probe amplification. Whole transcriptome sequencing identified a *SAMD12‐EXT1* fusion transcript that could have resulted from a chromosomal deletion, leading to the loss of *EXT1* function. Re‐review of the clinical array comparative genomic hybridization results indicated a possible unreported mosaic deletion affecting the *SAMD12* and *EXT1* genes that corresponded precisely to the introns predicted to be affected by a fusion‐causing deletion. The existence of the mosaic deletion was subsequently confirmed clinically by an increased density copy number array and orthogonal methodologies

**Conclusions:**

While mosaic mutations and deletions of *EXT1* and *EXT2* have been reported in the context of multiple osteochondromas, to our knowledge, this is the first time that transcriptomics technologies have been used to diagnose a patient via fusion transcript analysis in the congenital disease setting.

## 1 INTRODUCTION

Osteochondromas or exostoses are the most common form of benign bone tumor (Jennes et al., 2009; Schmale, Conrad, & Raskind, 1994). These cartilage‐capped outgrowths consist of a cortex and a marrow cavity that are continuous with the underlying bone. Approximately 15% of cases occur in the form of Hereditary Multiple Osteochondromas (HMO) and the disorder is inherited in an autosomal dominant manner, showing penetrance approaching 100% (Hennekam, 1991). Approximately 10% of HMO cases have no family history of the disorder and are believed to be caused by *de novo* mutations (Jennes et al., 2009).

Linkage analyses aimed at identifying the underlying cause of HMO led to the identification of probable disease loci on chromosomes 8q and 11p and the homologous genes *EXT1* and *EXT2* (OMIM IDs 608177 and 608210) were later characterized at these locations (Ahn et al., 1995; Stickens et al., 1996). Both genes are putative tumor suppressors which function as glucosyltranferases (McCormick et al., 1998).

Early estimates suggested that 70% of HMO cases carried mutations in either *EXT1* or *EXT2* (Philippe et al., 1997). Improved testing methodologies and increased profiling led to findings in 70%–95% of these cases (Wuyts & Van Hul, 2000), with *EXT1* mutated in approximately two‐thirds of cases and *EXT2* mutated in the remainder. HMO is highly heterogeneous from a mutation standpoint. Up to 77% of mutations have been shown to be private (Jennes et al., 2009) and published reports of novel variants continue to increase in number (Cao et al., 2014; Ciavarella et al., 2013; Faiyaz‐Ul‐Haque et al., 2004; Li et al., 2009; Pei et al., 2010; Sfar et al., 2009; Vanita, Sperling, Sandhu, Sandhu, & Singh, 2009; Wen et al., 2010).

Inactivating mutations such as nonsense, frameshift, or splice‐site mutations represent the majority of HMO causing mutations (75%–80%) (Jennes et al., 2009). While point mutations are the most frequently identified form of mutation, deletions involving single or multiple exons are found in up to 8% of cases and novel causative mechanisms have been identified in isolated instances (Waaijer et al., 2013).

EXT mutation testing usually consists of direct sequence analysis combined with methods such as fluorescence in situ hybridization (FISH), or multiplex ligation‐dependent probe amplification (MLPA) to maximize testing sensitivity (Jennes et al., 2011). In addition, high‐density copy number arrays revealed the existence of mosaic EXT mutations in 17% of individuals in a study of previously undiagnosed cases (Szuhai et al., 2011). Recently, exome sequencing and targeted next‐generation sequencing have been successfully employed in identifying further novel causative mutations while a single study reported diagnosis of aberrant *EXT1* splicing by sequencing blood‐derived RNA (Zhuang, Gerber, Kuchen, Villiger, & Trueb, 2016).

Further gene candidates including *EXT3* and others have been proposed to explain unresolved cases of HMO but none have been confirmed so far. It is highly likely that shortcomings in the molecular profiling of *EXT1* and *EXT2* account for numerous undiagnosed cases and that further refinement and expansion of testing methodologies may be required (Guo, Lin, Shi, Yan, & Chen, 2017; Hameetman et al., 2007; Jennes et al., 2009).

We describe the case of a patient with multiple phenotypic abnormalities including the presence of multiple osteochondromas. Initial testing for EXT mutations using several established clinical assays was negative, and the patient was eventually diagnosed by research‐based whole transcriptome sequencing. The case raises several issues relevant to EXT mutation profiling and wider genetic testing paradigms.

## 2 MATERIALS AND METHODS

### 2.1 Ethical compliance

This study was approved by the Mayo Clinic institutional review board and all participants provided written informed consent for genetic testing. The patient's family provided written consent for publication of identifiable images.

### 2.2 Study subjects and sample procurement

The proband was a male child who had been referred to Mayo Clinic's Center for Individualized Medicine in order to seek genetic diagnosis of a diverse phenotype. The patient and both parents underwent genetic counseling and a full case history and family pedigree were constructed. Blood samples were collected clinically from the proband and both unaffected parents to enable all subsequent genetic testing. DNA was isolated from blood samples using an Autopure LS automated DNA purifier (Qiagen) following the manufacturer's instructions. RNA was obtained by collecting blood in PAXgene blood RNA tubes and isolating using the QIAcube system (Qiagen), according to the manufacturer's protocol.

### 2.3 Chromosomal analysis

Chromosomal copy number analysis was performed by array comparative genomic hybridization (aCGH). Hybridization, washing, and analysis were performed according to the manufacturer's instructions (Agilent Technologies, Palo Alto, CA, USA). Oligonucleotide microarrays (44K and 180K arrays; Agilent Technologies) were of a uniform design developed through an academic laboratory consortium (Baldwin et al., 2008). Microarray hybridization data for each probe were computed with Agilent Feature Extraction software and analyzed using Agilent Genomic Workbench software (Agilent Technologies).

A laboratory‐developed FISH probe was utilized to confirm the 16p13.3 deletion (bacterial artificial chromosome (BAC) probe RP11‐698H1). Briefly, peripheral blood lymphocytes isolated from the proband and parents were cultured for 72 hr in PB‐Max plus excess thymidine and harvested according to standard cytogenetic protocols. Metaphases were dropped in a Thermotron chamber (Thermotron, Holland, MI, USA). Pre‐treatment and hybridization were performed according to standard cytogenetic protocols and metaphases were examined by fluorescence microscopy. Two different clinical cytogenetic technologists analyzed 5 metaphases each per patient (10 total per patient), and the results were interpreted by a clinical cytogeneticist.

### 2.4 Direct sequencing and MLPA Analysis for EXT1 and EXT2

DNA from a patient peripheral blood sample was used for direct sequencing and MLPA analysis of both *EXT1* and *EXT2* (Schouten et al., 2002). DNA analysis was performed by PCR‐based enrichment with previously described primers (Wuyts et al., 1998) followed by Sanger sequencing of all coding exons of the *EXT1* and *EXT2* genes and MLPA analysis (P215‐B3 kit, MRC‐Holland). GenBank entries NM_000127.2 (*EXT1*) and NM_207122.1 (*EXT2*) were used as reference sequences for mutation detection and reporting. *EXT2* exon numbering was according to a previously published gene model (Clines, Ashley, Shah, & Lovett, 1997). Classification of detected sequence variants was performed according to the 5‐class system: benign (class 1), likely benign (class 2), unknown clinical significance (class 3), likely pathogenic (class 4), pathogenic (class 5, mutation).

### 2.5 Exome sequencing and variant calling

DNA isolated from proband, maternal, and paternal peripheral whole blood was enriched using the Agilent SureSelect V5 exome capture kit. Paired‐end 101bp reads were generated to a depth of 20X across 97% of the capture region using an Illumina HiSeq 2500. Reads were aligned to the human genome (hg19) using Novoalign (Novocraft Technologies, Malaysia) followed by joint variant calling for the family trio using GATK HaplotypeCaller with PhaseByTransmission enabled (McKenna et al., 2010). Phased germline variants (SNVs and INDELs) were categorized in accordance with American College of Medical Genetics (ACMG) guidelines (Richards et al., 2015) and evaluated for clinical relevance by a multidisciplinary team of clinicians and researchers with expertise in genetics, genomics, and bioinformatics.

### 2.6 Whole transcriptome RNA sequencing

Proband RNA was isolated from peripheral whole blood and a sequencing library was prepared with the TruSeq RNA Sample Prep Kit v2 (Illumina, San Diego, CA, USA). The flow cells were sequenced as 100‐basepair paired‐end reads on an Illumina HiSeq 2500 using TruSeq Rapid SBS sequencing kit version 1 and HCS version 2.0.12.0 data collection software. Base calling was performed using Illumina's RTA version 1.17.21.3.

### 2.7 RNA fusion analysis

Candidate fusions were detected using raw outputs from Tophat Fusion (Kim & Salzberg, 2011) and false positives reduced using an internally formulated filtering cascade (Figure S1). In brief, fusion candidates were aligned to the human genome using BLAST (Altschul, Gish, Miller, Myers, & Lipman, 1990). Candidates corresponding to abundant hematological products (Globins, T‐cell receptors) were filtered. If a candidate contained sequence of unknown origin or produced an unbroken alignment against a known human sequence, it was removed from consideration. Candidates with low read support were also filtered. To control for events that might constitute common biological occurrences or recurrent artifacts, we compared our identified fusion candidates to a database of fusion events generated from normal samples (approximately 800 individuals/30 tissue types including whole blood) originating from within our own institution, and the GTEx project (Carithers et al., 2015). Fusion candidates were removed from consideration if they were identified at least two supporting reads in one or more control individuals. Candidate fusions were annotated based on Ensembl gene models (Zerbino et al., 2017) to identify gene partners, coding frame status, and exon–intron composition.

### 2.8 Molecular inversion probe (MIP) analysis for EXT mutations and copy number

Selection of *EXT1* and *EXT2* sequences was performed with molecular inversion probes targeted to all exons of *EXT1* and *EXT2*. Every position was covered by at least two different probes. Next‐generation sequencing analysis was performed on NextSeq500 sequencer (Illumina). Sequence analysis and CNV calling was performed using the SeqNext module of SequencePilot software (JSI medical systems).

## 3 RESULTS

### 3.1 Case presentation

The patient is a 9‐year‐old male child born at full term following an uncomplicated pregnancy and planned caesarian section. The patient was the second child born of a second pregnancy to a 31‐year‐old mother. Birthweight was 6lbs 12oz and both physical examination and Minnesota newborn screening (Minnesota Department of Health Newborn Screening Program) were normal. The patient followed the tenth percentile for weight and height for the first two months of life, before dropping off the curve below the third percentile while remaining normocephalic. At 6 months, the patient was diagnosed with failure to thrive. Failure to roll over or sit unaided led to a diagnosis of developmental delay. Recurrent acute otitis media led to the placement of myringotomy tubes at 10 months. Laryngoscopy, GI endoscopy, and swallow study were performed at the same age due to continued poor growth and all were normal. Extensive metabolic evaluations were also negative. A gastrostomy was performed at the age of 13 months and the patient made slow gains while remaining close to the third percentile of the growth chart.

The patient was unable to roll over until 7 months of age and did not sit or raise to his knees independently until 15 months. By 2 years, he could transfer objects between hands but retained difficulty in reaching for smaller objects. He could crawl and cruise unassisted by 18 months but did not walk independently until he was 3 years of age. Gait was noted to be wide and slightly unsteady with some toe‐walking and a tendency for tripping. Hyperreflexia of the patella was noted, as well as presence of some beats of clonus. At the age of 5 years, significant delays in receptive and expressive language were present, with an equivalency of 16 months.

**Figure 1:**
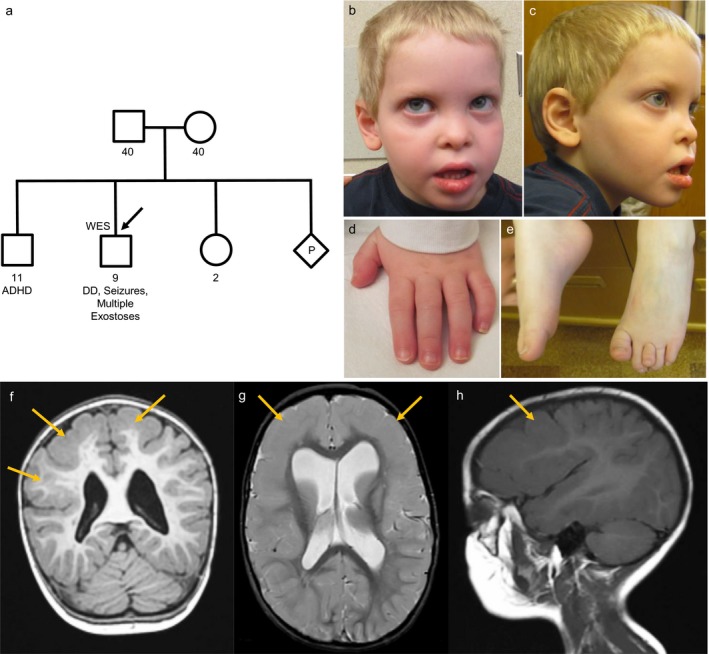
Family pedigree (a), photography (b–e), and (f–h) MRI images of the proband. Family pedigree was phenotypically unremarkable, whereas the proband manifested a phenotype including developmental delay, seizures, facial dysmorphisms, brachydactyly, and clinodactyly. (f–h) Magnetic resonance imaging of the brain revealed diffuse thickening of the cortical gray matter of both cerebral hemispheres and particularly prominent over the convexities. The junction of gray matter and white matter adjacent to this thickened cortex is smooth in contour, and there is evidence of thin curvilinear area within the thickened cortical gray matter that likely correlates with a dilatation of the lateral ventricles, particularly of the frontal horns. (f) MPRAGE coronal OBL (g) Axial OBL FSE T2 (h) Sagittal T1

**Figure 2:**
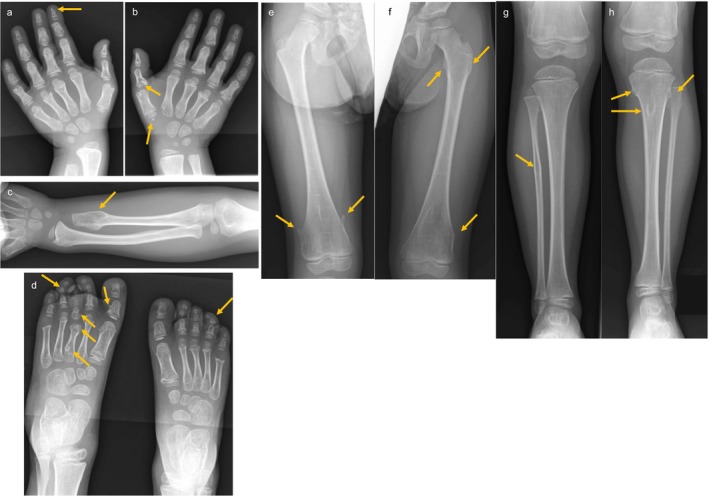
Radiographs of proband. (a,b) Multiple bilateral metacarpal and phalangeal osteochondromas. (c) Sessile protrusion on left ulna. (d) Multiple bilateral metatarsal outgrowths. (e) Pedunculated protrusions on distal left femur. (f) Outgrowths on proximal and distal right femur. (g) Suspected osteochondroma on proximal right fibula. (h) Outgrowths on left proximal tibia and fibula

The patient was evaluated by a medical geneticist and several dysmorphic features were noted including prominent forehead with slight frontal bossing, mild hypertelorism, prominent eyes, grayish sclera, short, low‐positioned nose with prominent columella and hypoplastic alae nasi (Figure 1b,c). There was mild shortening of the distal phalanges as well as mild overlap of second over third and fifth over fourth digits of the left hand (Figure 1d). The thumbs were slightly widened. Feet were normal except for slight upward displacement of the third toe of the left foot (Figure 1e). A skeletal survey was ordered and revealed a benign lucency in the proximal right fibular diaphysis with a pedunculated osteochondroma extending medially. A probable second osteochondroma was observed in the distal aspect of the left small finger proximal phalanx (Figure 2). Several wormian bones were noted along the right lambdoid suture.

At the age of 19 months, the patient suffered two febrile seizures. Four months later he had a series of non‐febrile seizures and was diagnosed with symptomatic localization‐related epilepsy by electroencephalogram (EEG) following status epilepticus. Magnetic resonance imaging (MRI) of the brain revealed diffuse bilateral frontotemporal pachygyria and dilation of the lateral ventricles (Figure 1f–h). The seizures were completely controlled by levetiracetam treatment.

Further skeletal evaluation at 5 years of age revealed the appearance of new osteochondromas including small sessile protrusions on the proximal left humerus and the proximal and distal femurs as well as left tibia and fibula and ulna. Multiple bilateral metatarsal, metacarpal, and phalangeal osteochondromas were also noted (Figure 2).

### 3.2 Family history

The patient was the second male child born to non‐consanguineous parents of mixed European descent (Figure 1a). The patient's mother was diagnosed as asthmatic and did not speak until 3 years of age. A male maternal cousin also had a speech delay. The patient's father and paternal uncle were both short of stature and low weight as children. The patient's older brother was diagnosed with attention deficit hyperactivity disorder (ADHD) but had normal speech and no issues with ambulation. A younger sister and both maternal and paternal grandparents were phenotypically unremarkable.

### 3.3 Chromosomal analysis

Chromosomal analysis revealed a normal 46,XY karyotype with no visible irregularities. Array comparative hybridization (aCGH) conducted clinically yielded equivocal results (Agilent 44k array). An interstitial deletion of approximately 4 oligonucleotide probes at 16p13.3 spanning 219 kilobases was observed (arr 16p13.3(2576112_2794743)x1 [hg18]). Metaphase FISH analysis confirmed the presence of the deletion (BAC probe RP11‐698H1) while parental FISH studies demonstrated that the deletion was not inherited from either parent and was therefore likely a *de novo* event. The deleted interval contained eleven known genes including *PDPK1*,* LOC652276*,* FLJ42627*,* ERVK13‐1*,* KCTD5*,* PRSS27*,* SRRM2‐AS1*,* SRRM2*,* TCEB2*,* PRSS33*, and *PRSS41*. None of the eleven genes showed direct links to the patient's phenotype based on published works or online genotype–phenotype databases. A previous case report (Nelson, Quinonez, Ackley, Iyer, & Innis, 2011) describing a patient with multiple congenital abnormalities including global developmental delay, tracheobronchomalacia, and fifth finger clinodactyly had a partially overlapping 555 kb *de novo* 16p13.3 deletion (Figure S2). Since there was only partial phenotypic overlap, the deletion in our patient was reported as of uncertain significance.

### 3.4 Sequencing and MLPA for EXT1 and EXT2

The results reported by direct sequencing and MLPA analysis of *EXT1* and *EXT2* were negative suggesting the absence of point mutations or copy number alterations in the EXT genes.

### 3.5 Exome sequencing

Clinical exome analysis revealed the presence of a hemizygous, maternally inherited splice acceptor variant (ChrX(GRCh37): g.110574270C>T; NM_178153.2(*DCX*): c.809‐1G>A) in the Doublecortin gene (*DCX,* OMIM ID 300121). Doublecortin is involved in neuronal migration and *DCX* loss‐of‐function mutations are responsible for X‐linked lissencephaly in hemizygous males (MIM# 300067) with symptoms that include pachygyria, seizures, and delayed motor development. Heterozygous females are typically affected by subcortical laminal heterotopia and have a milder and incompletely penetrant phenotype which likely accounts for the absence of symptoms in the proband's mother. The *DCX* variant had previously been reported in the ClinVar database (RCV000145892.1) and was classified as likely pathogenic according to ACMG guidelines (Richards et al., 2015). Exome sequencing failed to reveal any variants of interest in *EXT1, EXT2*, or any other genes implicated in HMO. Three further variants in the *ARSE* and *FLG* genes were detected but classified as being of unknown clinical significance (Table 1).

**Table 1:** Variants detected by clinical exome sequencing

Gene	Transcript	Variant	Location	Zygosity	Origin	Classification
*DCX*	NM_178153.2	809‐1G>A	chrX:110574270	Hemizygous	Maternal	Likely pathogenic
*ARSE*	NM_000047.2	1694T>G (Ile565Ser)	chrX:2852949	Hemizygous	Maternal	Unknown significance
*FLG*	NM_002016.1	7801G>A (Asp2601Asn)	chr1:152279561	Heterozygous	Maternal	Unknown significance
*FLG*	NM_002016.2	2379_2394delinsTCCTCAG (Leu794_Ser798delinsProGln)	chr1:152284968_152284983	Heterozygous	Paternal	Unknown significance

### 3.6 Whole transcriptome sequencing

**Figure 3:**
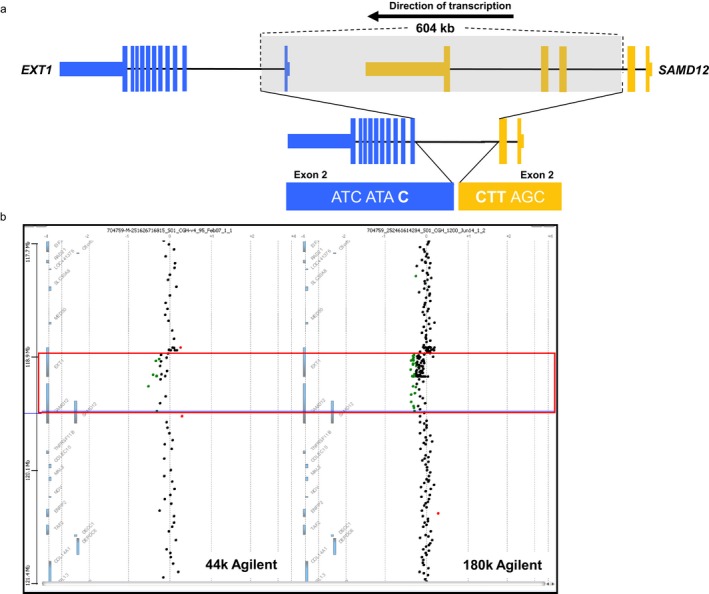
Formation of a *SAMD12‐EXT*1 fusion transcript (a) and (b) confirmation by high‐density aCGH in a patient diagnosed with multiple osteochondromas. (a) A 604 KB deletion of chromosome 8 leads to joining of the genomic sequence within *SAMD12* intron 2 and *EXT1* intron 1. The transcriptional product formed post‐splicing is a fusion transcript consisting of the 3` portion of *SAMD12* (Exons 1‐2) fused to the 5` portion of *EXT1* (Exons 2‐11). The two native transcripts are joined within their coding regions bringing about an interruption of the triplet codon reading frame, corresponding to a loss‐of‐function. The fusion transcript was detected by RNA‐Seq while the underlying mosaic deletion was identified using a combination of Agilent 44k and 180k copy number arrays. (b) Confirmation of a 604 KB chromosome 8 deletion causing formation of a *SAMD12*‐*EXT1* fusion transcript in a patient diagnosed with multiple osteochondromas. Initial copy number analysis was performed with an Agilent 44k array (left panel) but the mosaic deletion (red box) underlying the fusion event went undetected as it fell below clinical reporting thresholds. Following detection of the candidate *SAMD12‐EXT1* fusion transcript using RNA‐Seq, clinical results originally below reporting threshold were investigated and revealed the mosaic genomic deletion. Follow‐up confirmation was performed using an increased density Agilent 180k array (right panel). The deletion boundaries correspond to intronic positions that are ultimately joined, before the processes of transcription and intronic splicing bring about the mature fusion transcript consisting of Exons 1‐2 of *SAMD12* and Exons 2‐11 of *EXT1*

The patient was included in a pilot study to assess the utility of whole transcriptome RNA sequencing in undiagnosed rare disease. Profiling the RNA‐Seq data for the presence of fusion transcripts revealed 17 reads supporting the existence of a *SAMD12‐EXT1* intrachromosomal fusion candidate (Figure S3). The putative event involved the fusion of the 3` boundary of *SAMD12* exon 2 (chr8:119592952) to the 5` boundary of *EXT1* exon 2 (chr8:118849438) (Figure S4). The event was not identified in an internally generated normal sample fusion control database. RPKM values for *SAMD12* and *EXT1* in the patient sample were 0.124 and 0.85, respectively, corresponding to peak read depths of 75 and 167 for the exons involved in the fusion. Twelve of the 17 supporting reads physically crossed the *SAMD12:EXT1* exon:exon boundary, whereas 167 reads representing the normal transcripts crossed the *EXT1* exon 2:exon 3 boundary and 22 reads crossed the *SAMD12* exon2:exon3 boundary. *SAMD12* (OMIM ID 618073) lies approximately 20 kb upstream of *EXT1* on the reverse strand of chromosome 8. The orientation of the genes in the fused transcript is in agreement with their direction of transcription on chromosome 8, which led to the hypothesis that a chromosomal deletion of the genomic region between *SAMD12* exon 2 and *EXT1* exon 2 might account for the occurrence of the fusion transcript. Both genes were joined within their coding sequence, and the fusion was predicted to cause an interruption of the reading frame, equivalent to loss‐of‐function of both genes (Figure 3a). A second fusion candidate was observed corresponding to the breakpoints of the 16p13.3 deletion detected by aCGH (Figure S5) while three further fusion candidates were unsupported by aCGH (Table 2).

**Table 2:** Candidate fusion transcripts detected from RNA‐Seq

Fusion partners	Fused location	Predicted in‐frame?	Reads supporting fusion	5` Chr	5` Pos	3` Chr	3` Pos	Supported by aCGH?
*SRP54‐AS1 ‐ BAZ1A*	Exon–Exon boundary	No	6	chr14	35433135	chr14	35343868	NO
*PDPK1‐PRSS21*	Exon–Exon boundary	No	51	chr16	2633586	chr16	2875971	YES
*MNT‐METTL16*	Exon–Exon boundary	No	43	chr17	2297336	chr17	2317764	NO
*SAMD12‐EXT1*	Exon–Exon boundary	No	17	chr8	119592952	chr8	118849438	YES
*GSR‐PPP2CB*	Exon–Exon boundary	Yes	9	chr8	30585047	chr8	30657271	NO

### 3.7 MIP analysis for EXT mutations and copy number detection

NGS sequencing after MIP enrichment showed no pathogenic variant in the *EXT1* or *EXT2* gene in agreement with the Sanger sequencing results. However, copy number analysis indicated the presence of an *EXT1* exon 1 deletion (Figure S6).

### 3.8 Clinical follow‐up

To assess the possibility of a previously unidentified copy number loss in the genomic region between *SAMD12* and *EXT1,* the MLPA and aCGH results were re‐reviewed for the possibility of a sub‐calling threshold event. Raw MLPA results were checked for any indication of a deletion of *EXT1* exon 1. However, it was impossible to draw any conclusion about a possible deletion. The EXT MLPA analysis was repeated and resulted again in a negative result with default analysis settings (cutoff deletion ratio 0.75). However, the two *EXT1* exon 1 probes showed a ratio of 0.82 and 0.87 while all other *EXT1* probes showed a ratio of 0.97 or higher, a result compatible with a possible mosaic deletion. Reassessment of the clinical aCGH revealed 12 consecutive probes within the region of interest that were identified as having a modestly reduced intensity/log ratio (mean log_2_ ratio of −0.229) (Figure 3b). It was theorized that this region of lowered probe intensities may be indicative of a mosaic loss in the genomic region between the fused exons. A higher density copy number array (Agilent 180k array) was run for confirmation. In this case, 53 consecutive probes in the suspected region were identified as having reduced probe intensities corresponding to a mean log_2_ ratio of −0.206 and reinforcing the previous aCGH result, consistent with a mosaic deletion (Figure 3b). The deletion was classified as mosaic due to the results of the two gold‐standard aCGH results. The mosaic loss was further supported by the single positive MLPA result and a *post hoc* analysis of exon coverage based on exome sequencing data (results not shown). The higher amplitude loss suggested by MIP analysis likely reflects needs for further optimization of this in‐development assay. Based on the probe locations of the increased density array, the deleted region was estimated to be 604 kb in size, occurring between chr8:118960168‐119569348. This deletion corresponded precisely to positions within *SAMD12* intron 2 and *EXT1* intron 2. The result of such a deletion post transcription and splicing would be the observed fusion of exons 2 of *SAMD12* and *EXT1,* causing loss‐of‐function of *EXT1*. The validated fusion was classified as pathogenic and diagnostic of the patient's HMO.

## 4 DISCUSSION

We report the first known case of gene fusion as a mechanism of *EXT1* loss‐of‐function in HMO and the second case of an aberrant *EXT1* transcript detected from blood‐derived RNA (Zhuang et al., 2016). While fusion transcripts are routinely profiled in tumor studies they remain under‐characterized in congenital disorders, despite multiple published examples of their detection and diagnostic utility (Backx, Seuntjens, Devriendt, Vermeesch, & Van Esch, 2011; Ceroni et al., 2014; Hackmann et al., 2013; Holt et al., 2012; Mayo et al., 2017; Moysés‐Oliveira et al., 2015; Ramocki et al., 2003). A few recent studies have begun to promote the routine use of transcriptomic assays to aid in the diagnosis of inherited disease for which causal variants remain elusive (Cummings et al., 2017; Kremer, Wortmann, & Prokisch, 2018) and have demonstrated significant gains in diagnostic yield. This study adds to the growing number of fusion transcripts diagnostic of inherited disease and further reinforces the position of RNA‐Seq as a companion assay when standard testing fails to reveal a diagnostic event. Ultimately, we hope that research‐based RNA‐Seq methodologies transition increasingly toward routine clinical use.

Despite its utility, RNA‐Seq analysis in the rare disease setting presents analytical challenges. Fusion analyses can initially generate thousands of false‐positive candidate events, and formulation of sensitive and specific filtering cascades is non‐trivial. The validation of our approach is the small number of candidate events that required manual review (5 in this case) and the ultimate discovery of two fusion events, each verified by clinical aCGH. We continue to pursue validation of fusion events in individuals with undiagnosed diseases (Cousin et al., 2018) and believe that sustained application of these methods will lead to resolution of further unsolved cases.

While novel, the discovery of a *SAMD12‐EXT1* fusion is in keeping with prior reports of heterogeneous events underlying EXT inactivation (Jennes et al., 2009; Waaijer et al., 2013). Large deletions, including whole‐gene losses are reported in approximately 5% of HMO cases (Jennes et al., 2008, 2009; Pedrini et al., 2011) and these are generally reported to be unique events. The largest reported study of precise breakpoints in *EXT1* deletions (Jennes et al., 2011) reported solely non‐recurrent events while The Multiple Osteochondromas Mutation Database (Jennes et al., 2009) and several other studies report largely non‐recurring *EXT1* deletions whose breakpoints are distinct from our own (Jennes et al., 2008; Li, Wang, Wang, Tang, & Yu, 2017; Santos et al., 2018; White et al., 2004; Zhuang et al., 2016). Deletions of *EXT1* exon 1 are reported recurrently but we could not identify any previously reported deletion that corresponded to the breakpoints detected in this study.

Notably, the *SAMD12‐EXT1* fusion in our patient is not the first observed fusion event between these genes. The Cancer Genome Anatomy Project and The Cancer Genome Atlas report a small number of *EXT1‐SAMD12* fusions observed in tumor samples including oral, breast and head and neck tumors (http://AtlasGeneticsOncology.org). The reversed juxtaposition of the genes in these events relative to our own suggests an alternative underlying mechanism but they are similarly predicted to cause loss‐of‐function. Nonetheless, their genetic origin and biological relevance remain unclear.

It is also worth considering the likely attenuation of *SAMD12* function. *SAMD12* disease‐relevance is not well characterized but recent publications have reported an intronic heterozygous *SAMD12* pentanucleotide repeat insertion causative of familial cortical myoclonic tremor with epilepsy type 1 (Ishiura et al., 2018). While the proband is unaffected by myoclonus, it could be hypothesized that the mechanistically distinct, mosaic loss‐of‐function observed in this case could elicit a unique effect with relevance the observed phenotype. Ultimately however, either functional validation studies or further patient‐based analyses are necessary to draw any conclusion and *SAMD12* represents a gene whose functional links should be flagged for future systematic reanalysis (Hiatt et al., 2018; Wenger, Guturu, Bernstein, & Bejerano, 2017).

A further consideration is the clinically reported 16p13.3 deletion and corresponding detection of a *PDPK1‐PRSS21* fusion transcript. The fusion transcript provides an intriguing alternate viewpoint and revalidation of the deletion event. The fused exon boundaries offer a high‐resolution view of the precise effect of the underlying deletion where array probes lack density. Further, the traditional approach to consideration of chromosomal deletions in genetic disease is to assume loss‐of‐function of the interstitial genes, but it is likely that the genes bounding a deletion event should be routinely assessed for potential gene fusions.

This case report raises several issues of relevance to the clinical assessment of HMO patients. Our findings support diversified EXT profiling in HMO cases where initial testing fails to identify a causal mutation. Further, the mosaic nature of the deletion underlying the *SAMD12‐EXT1* is consistent with previous reports of mosaicism underlying unsolved HMO cases (Sarrión et al., 2013; Szuhai et al., 2011). Our findings also suggest that aCGH of peripheral blood samples is a viable method for broader identification of mosaic deletion events. However, it appears paramount that reporting thresholds be evaluated and cases previously classified as negative be reinspected for potential missed events. *EXT1*‐targeted FISH analysis can be used to increase diagnostic rates in case of mosaic events, but it is unlikely to have possessed sufficient sensitivity to help in this case where only a single *EXT1* exon was deleted. While combined direct sequencing and MLPA is widely considered the gold‐standard technique for EXT mutation profiling, a negative result should likely trigger follow‐up analysis with high‐density aCGH rather than the pursuit of variation in novel genes. Alternatively, the use of next‐generation sequencing based copy number analysis may be more sensitive first‐line screening strategy. With every exon covered by several MIP probes, this design shows increased sensitivity to detect mosaic copy number variations compared to MLPA. Conversely, a retrospective copy number analysis of the proband's exome sequencing data using a published algorithm (Wang et al., 2014) was able to detect the mosaic deletion of the affected exons of *SAMD12* and *EXT1*, achieving a log ratio comparable to that detected by aCGH (data not shown). This finding offers some promise for the application of exome sequencing for simultaneous variant detection and copy number analysis.

A separate consideration raised here is the issue of multi‐genic diagnoses. Whole exome sequencing is now routinely applied in studies of undiagnosed disease, yielding a broad profile of variation across most known genes. It is becoming increasingly apparent that diverse or multisystem phenotypes may justify further investigation beyond an initial confirmed pathogenic variant (Posey et al., 2017). In the context of the current patient, the presentation of multiple osteochondromas prevented the case being classified as solved upon detection of the partially diagnostic *DCX* variant but a less distinct phenotypic component may have avoided further investigation.

In summary, we have described a diagnostic odyssey case where clinical exome sequencing was able to yield a partial diagnosis but this and further gold‐standard multi‐assay clinical testing failed to resolve an unexplained osteochondroma phenotype due to a mosaic event. A complete diagnosis was only achieved through a combination of extensive testing and reflexive analysis of unreported clinical results. This finding has relevance for both EXT and wider diagnostic odyssey testing.

## CONFLICT OF INTEREST

The authors of this work have no conflicts of interest to declare.
