# Supplementary material for: RNA‐Seq detects a SAMD12‐EXT1 fusion transcript and leads to the discovery of an EXT1 deletion in a child with multiple osteochondromas
Source: Mol Genet Genomic Med. 2019 Jan 10;7(3):e00560. doi: 10.1002/mgg3.560 (PMC6418362; doi:10.1002/mgg3.560)

Supplementary Figure 1

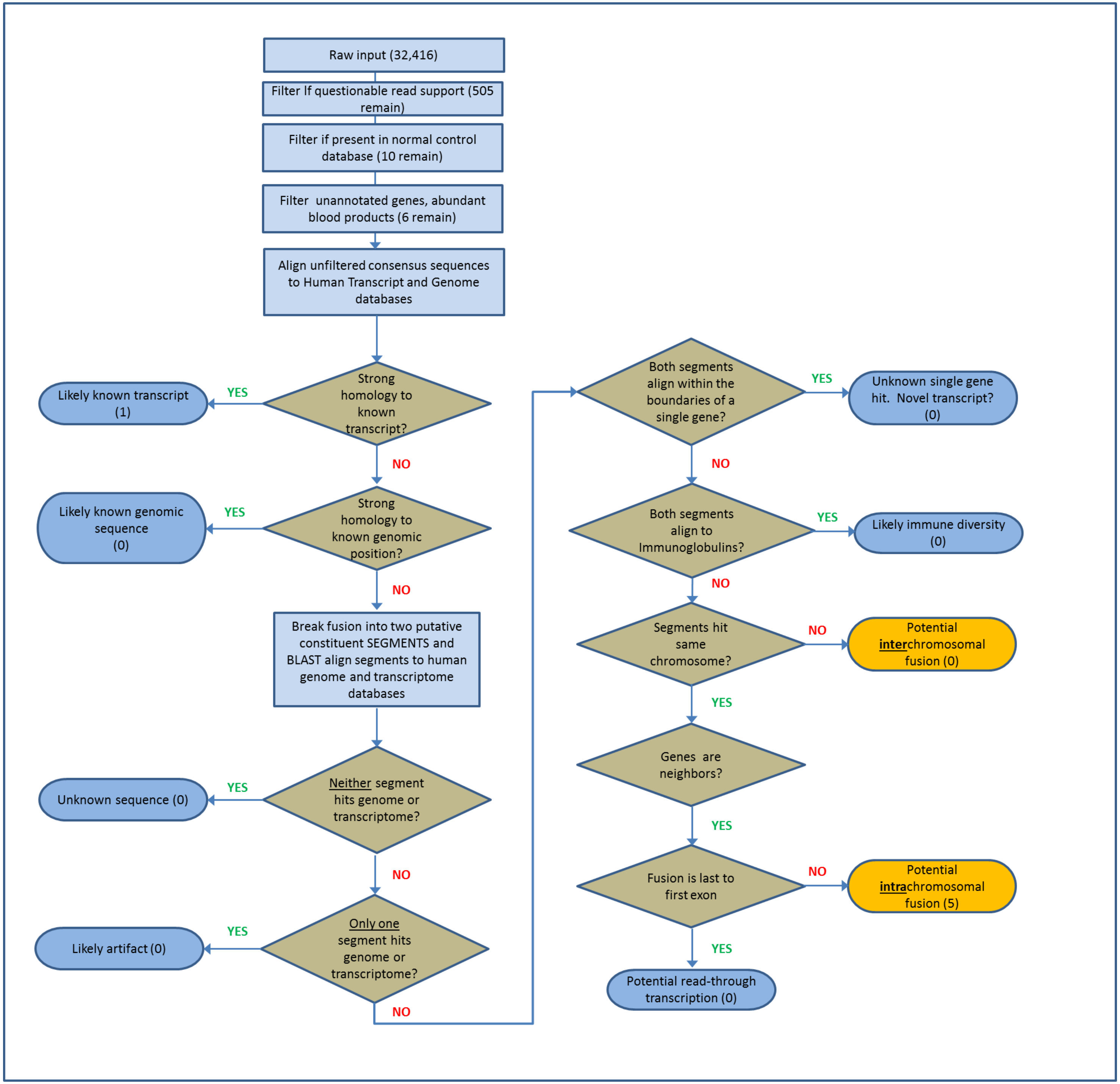

Supplementary Figure 2

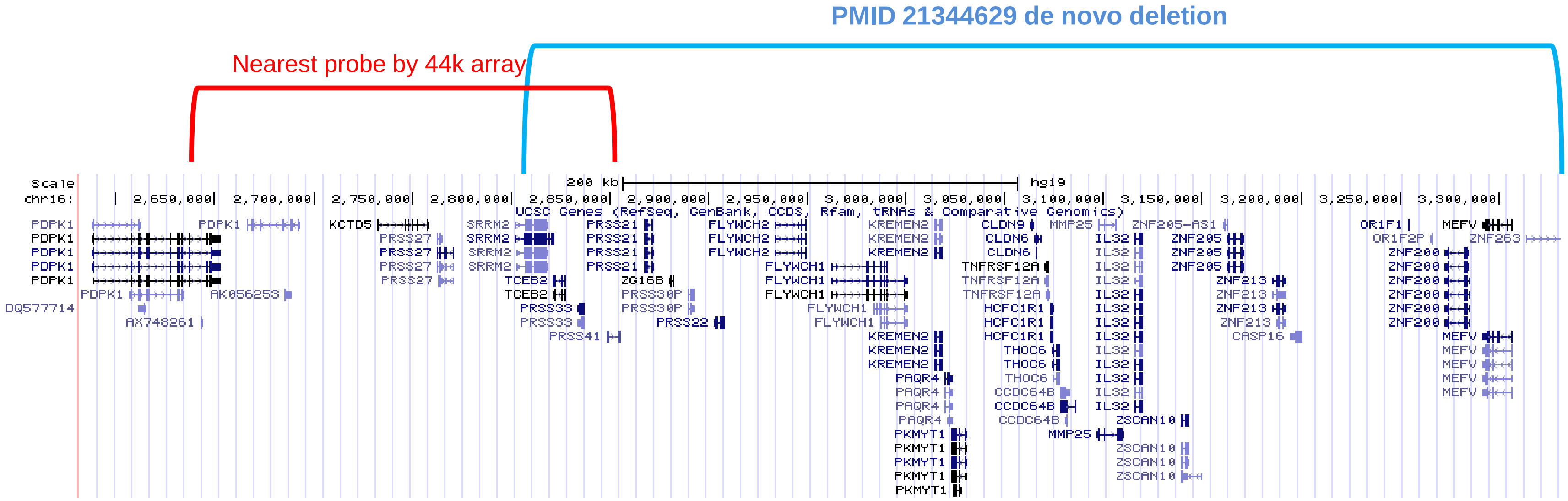

Supplementary Figure 3

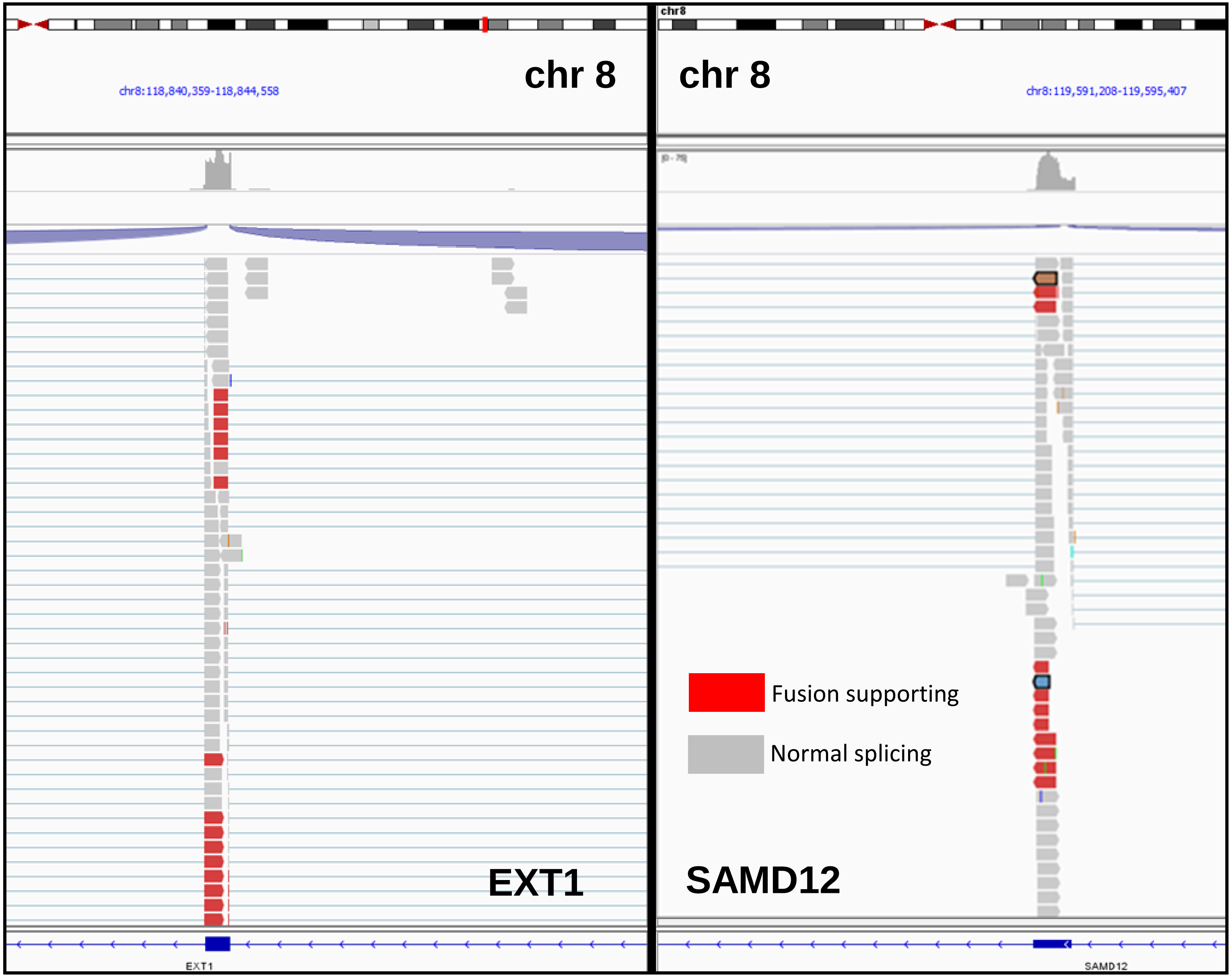

Supplementary Figure 4

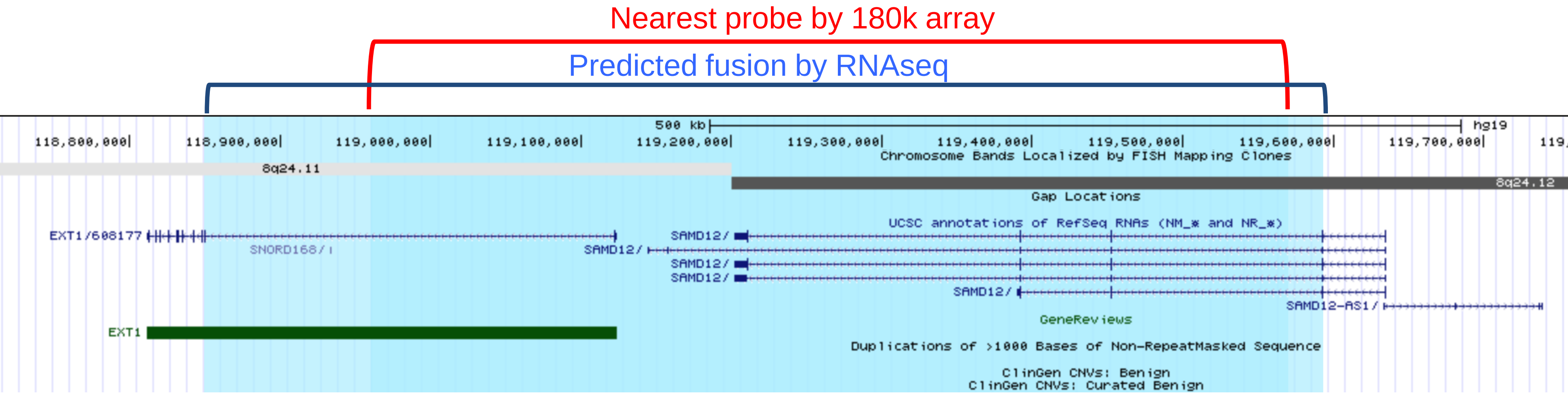

Supplementary Figure 5

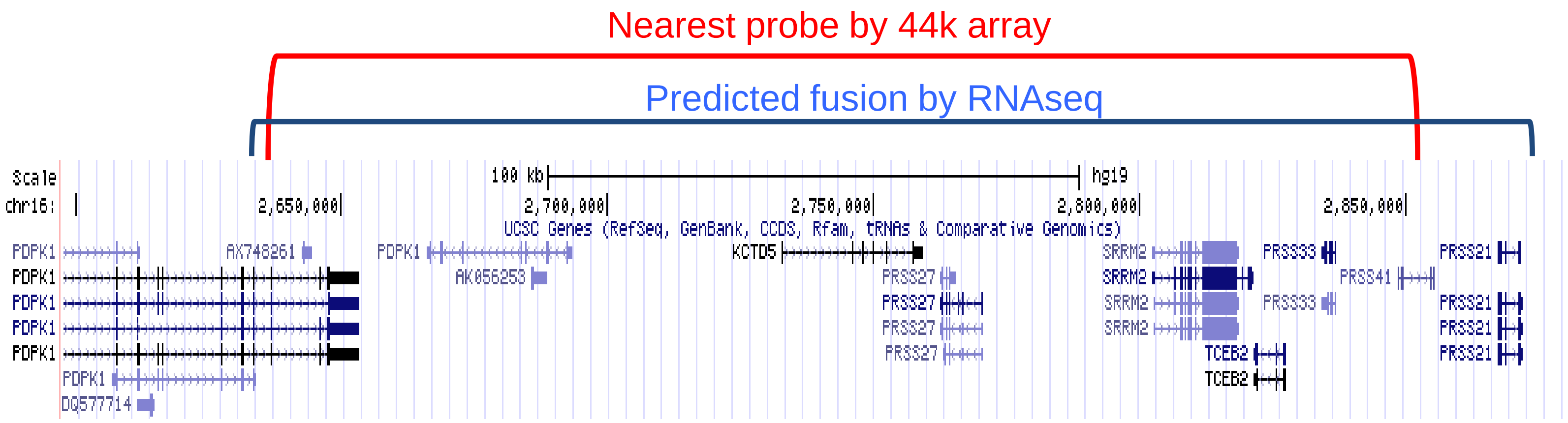

Supplementary Figure 6

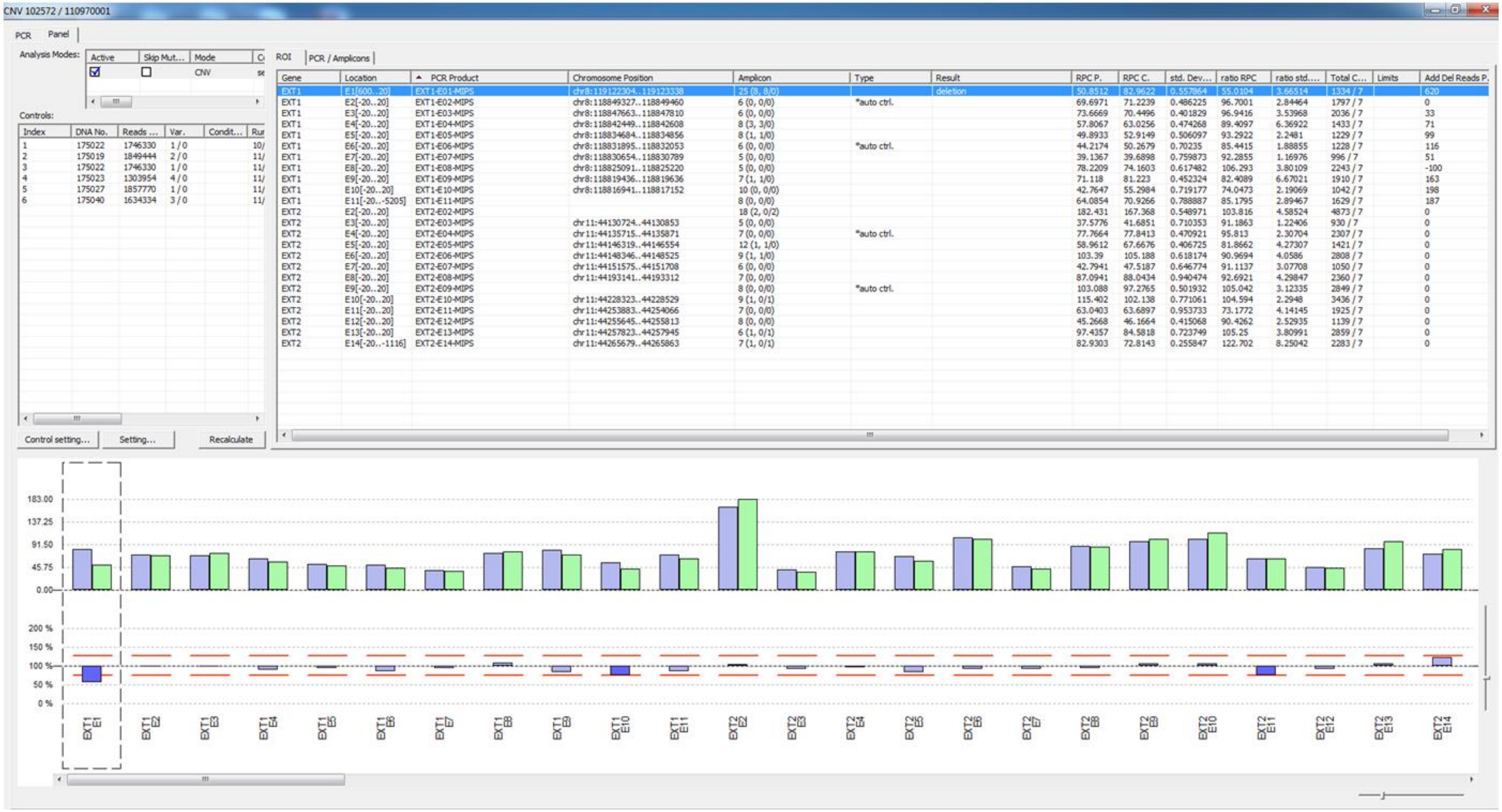

Supplement: Supplementary file 1 [file MGG3-7-na-s001.pdf]
